# Consolidated Reporting Guidelines for Prognostic and Diagnostic Machine Learning Models (CREMLS)

**DOI:** 10.2196/52508

**Published:** 2024-05-02

**Authors:** Khaled El Emam, Tiffany I Leung, Bradley Malin, William Klement, Gunther Eysenbach

**Affiliations:** 1 School of Epidemiology and Public Health University of Ottawa Ottawa, ON Canada; 2 Children’s Hospital of Eastern Ontario Research Institute Ottawa, ON Canada; 3 JMIR Publications, Inc Toronto, ON Canada; 4 Department of Internal Medicine (adjunct) Southern Illinois University School of Medicine Springfield, IL United States; 5 Department of Biomedical Informatics Vanderbilt University Nashville, TN United States; 6 School of Health Information Science University of Victoria Victoria, BC Canada

**Keywords:** reporting guidelines, machine learning, predictive models, diagnostic models, prognostic models, artificial intelligence, editorial policy

## Abstract

The number of papers presenting machine learning (ML) models that are being submitted to and published in the *Journal of Medical Internet Research* and other JMIR Publications journals has steadily increased. Editors and peer reviewers involved in the review process for such manuscripts often go through multiple review cycles to enhance the quality and completeness of reporting. The use of reporting guidelines or checklists can help ensure consistency in the quality of submitted (and published) scientific manuscripts and, for example, avoid instances of missing information. In this Editorial, the editors of JMIR Publications journals discuss the general JMIR Publications policy regarding authors’ application of reporting guidelines and specifically focus on the reporting of ML studies in JMIR Publications journals, using the Consolidated Reporting of Machine Learning Studies (CREMLS) guidelines, with an example of how authors and other journals could use the CREMLS checklist to ensure transparency and rigor in reporting.

## Introduction

The number of papers presenting machine learning (ML) models that are being submitted to and published in the *Journal of Medical Internet Research* and other JMIR Publications journals has steadily increased over time. The cross-journal JMIR Publications e-collection “Machine Learning” includes nearly 1300 articles as of April 1, 2024 [1], and there are additional sections in other journals, which collate articles related to the field (eg, “Machine Learning from Dermatological Images” [2] in *JMIR Dermatology*). From 2015 to 2022, the number of published articles with “artificial intelligence” (AI) or “machine learning” in the title and abstract in JMIR Publications journals increased from 22 to 298 (13.5-fold growth), and there are already 312 articles in 2023 (14-fold growth). For *JMIR Medical Informatics*, the number of articles increased from 10 to 160 (16-fold growth) until 2022. This is consistent with the growth in the research and application of medical AI in general where a similar PubMed search (with the keyword “medicine”) revealed a 22-fold growth (from 640 to 14,147 articles) between 2015 and 2022, and there are already 11,272 matching articles in 2023.

Many papers reporting the use of ML models in medicine have used a large clinical data set to make diagnostic or prognostic predictions [3-6]. However, the use of data from electronic health records and other resources is often not without pitfalls as these data are typically collected and optimized for other purposes (eg, medical billing) [7].

Editors and peer reviewers involved in the review process for such manuscripts often go through multiple review cycles to enhance the quality and completeness of reporting [8]. The use of reporting guidelines or checklists can help ensure consistency in the quality of submitted (and published) scientific manuscripts and, for instance, avoid instances of missing information. For example, in the experiences of the editors-in-chief of *JMIR AI*, missing information is especially notable because for manuscripts reporting on ML models, which are submitted to *JMIR AI*, this can delay the overall review interval by adding more revision cycles.

According to the EQUATOR (Enhancing the Quality and Transparency of Health Research) network, a reporting guideline is “a simple, structured tool for health researchers to use while writing manuscripts. A reporting guideline provides a minimum list of information needed to ensure a manuscript can be, for example: understood by a reader, replicated by a researcher, used by a doctor to make a clinical decision, and included in a systematic review” [9]. These can be presented in the form of a checklist, flow diagram, or structured text.

In this Editorial, we discuss the general JMIR Publications policy regarding authors’ application of reporting guidelines. We then focus specifically on the reporting of ML studies in JMIR Publications journals.

## JMIR Publications Policy on the Use of Reporting Guidelines

Accumulating evidence suggests that when authors apply reporting guidelines and reporting checklists in health research, they can be beneficial for authors, readers, and the discipline overall by enabling the replication or reproduction of studies. Recent evidence suggests that asking reviewers to use reporting checklists, instead of authors, offers no added benefits regarding reporting quality [10]. However, Botos [11] reported a positive association between reviewer ratings of adherence to reporting guidelines and favorable editorial decisions, while Stevanovic et al [12] reported a significant positive correlation between adherence to reporting guidelines and citations and between adherence to reporting guidelines and publication in higher-impact-factor journals.

JMIR Publications’ editorial policy recommends that authors adhere to applicable study design and reporting guidelines when preparing manuscripts for submission [13]. Authors should note that most reporting guidelines are strongly recommended, particularly because they can improve the quality, completeness, and organization of the presented work. At this time, JMIR Publications *requires* reporting checklists to be completed and supplied as multimedia appendices for randomized controlled trials without [14-16] or those with eHealth or mobile health components [17], systematic and scoping literature reviews across the portfolio, and Implementation Reports in *JMIR Medical Informatics* [18]. Although some medical journals have mandated the use of certain reporting guidelines and checklists, JMIR Publications recognizes that authors may have concerns about the additional burden that the formalized use of checklists may bring to the submission process. As such, JMIR Publications has chosen to begin recommending the use of ML reporting guidelines and will evaluate their benefits and gather feedback on implementation costs before considering more stringent requirements.

## Reporting on ML Models

Regarding the reporting of prognostic and diagnostic ML studies, multiple directly relevant checklists have been developed. Klement and El Emam [19] have consolidated these guidelines and checklists into a single set that we refer to as the Consolidated Reporting of Machine Learning Studies (CREMLS) checklist. CREMLS serves as a reporting checklist for journals publishing research describing the development, evaluation, and application of ML models, including all JMIR Publications journals, which have officially adopted these guidelines. CREMLS was developed by identifying existing relevant reporting guidelines and checklists. The initial item list was identified through a structured literature review and expert curation, and then the quality of the methods used for their development was assessed to narrow them down to a high-quality subset. This high-quality item subset was further filtered to reveal those that meet specific inclusion and exclusion criteria. The resultant items were converted to guidelines and a checklist that was reviewed by the editorial board of *JMIR AI*, followed by a preliminary application to assess articles published in *JMIR AI*. The final checklist offers present-day best practices for high-quality reporting of studies using ML models.

Examples of the application of the CREMLS checklist are presented in Table 1. In doing so, we identified 7 articles published in JMIR Publications journals, which exemplify each checklist item. Note that not all of the items are relevant to each article, and some articles are particularly good examples of how to operationalize a checklist item.

**Table 1:** Illustration of how various articles published in JMIR Publications journals implement each of the CREMLS (Consolidated Reporting of Machine Learning Studies) checklist items.

Item number			Item		Example illustrating the item
**Study details**					
	1.1	The medical or clinical task of interest		Examines chronic disease management—a clinical problem with 4 example solutions using ML^a^ models [20]	
	1.2	The research question		Proposes a framework to transfer old knowledge to a new environment to manage drifts [21]	
	1.3	Current medical or clinical practice		Provides a review of current practice and issues associated with chronic disease management [20]	
	1.4	The known predictors and confounders of what is being predicted or diagnosed		Describes variables defined as part of a well-established health test available to the public [20]	
	1.5	The overall study design		Presents experimental design with data flow and data partitions used at various steps of the experiment (Figure 1 [22])	
	1.6	The medical institutional settings		Describes the institution as an academic (teaching) community hospital where the data were collected [23]	
	1.7	The target patient population		Clear partitioning of target patient populations and the comparator group [20]	
	1.8	The intended use of the ML model		Describes how the prediction model fits in the clinical practice of scheduling operating theater procedures [5]	
	1.9	Existing model performance benchmarks for this task		Reviews existing research and presents achieved performance (eg, AUC^b^) [20]	
	1.10	Ethical and other regulatory approvals obtained		Ethics approvals [5]	
**The data**					
	2.1	Inclusion or exclusion criteria for the patient cohort		Defined in Figure 1 in the paper by Kendale et al [5]	
	2.2	Methods of data collection		Describes sources and methods of data collection, what type of data were used, and potential implied bias in interpretation [23]	
	2.3	Bias introduced due to the method of data collection used		Discusses potential bias in data collection and outcome definition [23]	
	2.4	Data characteristics		Uses descriptive statistics to show data characteristics for different types of data (demographics and clinical measurements) [23]	
	2.5	Methods of data transformation and preprocessing applied		Imputation is discussed [5]	
	2.6	Known quality issues with the data		Missingness and outlier detection were discussed [5]	
	2.7	Sample size calculation		Brief section dedicated to power analysis [5]	
	2.8	Data availability		Explains how to obtain a copy of the data [24]	
**Methodology**					
	3.1	Strategies for handling missing data		Describes how missing values were replaced [20]	
	3.2	Strategies for addressing class imbalance		Describes the approach of using SMOTE^c^ to adjust class ratios to address imbalance [23]	
	3.3	Strategies for reducing dimensionality of data		Describes the vectorization of a dimension of 100 into a 2D space using an established algorithm [22]	
	3.4	Strategies for handling outliers		The authors stated the threshold values used to detect outliers [5]	
	3.5	Strategies for data augmentation		Showed how variable similarity is achieved between synthetic and real data in the context of augmentation [24]	
	3.6	Strategies for model pretraining		Describes and illustrates (Figure 1) how models from other data sets were trained and used in the new model [23]	
	3.7	The rationale for selecting the ML algorithm		Discusses properties of the selected algorithm relevant to the problem at hand as motivation [20]	
	3.8	The method of evaluating model performance during training		Presents a separate discussion of evolution in cross-validation settings and external evaluation while also describing hyperparameter tuning [23]	
	3.9	The method used for hyperparameter tuning		Comprehensive description of tuning within nested cross-validation (this is a tutorial but illustrates how to describe the process) [25]	
	3.10	Model’s output adjustments		Describes the final model, how it was calibrated and discusses the impact of embedding on patient data for interpretation [22]	
**Evaluation**					
	4.1	Performance metrics used to evaluate the model		Comprehensive and detailed discussion of evaluation and quality metrics [24]	
	4.2	The cost or consequence of errors		Comprehensive error analysis [25]	
	4.3	The results of internal validation		Detailed validation discussion (internally and externally) [25]	
	4.4	The final model hyperparameters		Presents details of the final model and the winning parameters [5]	
	4.5	Model evaluation on an external data set		Detailed and comprehensive external validation that is separate from model testing [5]	
	4.6	Characteristics relevant for detecting data shift and drift		Implements performance monitoring, addresses data shifts over time, and illustrates them in detail [21]	
**Explainability and transparency**					
	5.1	The most important features and how they relate to the outcomes		Presents variable importance (SHAP^d^ values) in the context of interpretation and compares it to existing literature [5]	
	5.2	Plausibility of model outputs		Shows sample output (Figure 4 in the paper by Kendale et al [5])	
	5.3	Interpretation of a model's results by an end user		Good discussion about interpretability and use of the final model [5]	

^a^ML: machine learning.

^b^AUC: area under the curve.

^c^SMOTE: synthetic minority oversampling technique.

^d^SHAP: Shapely additive explanations.

We strongly advise authors who seek to submit their manuscripts describing the development, evaluation, and application of ML models to the *Journal of Medical Internet Research*, *JMIR AI*, *JMIR Medical Informatics*, or other JMIR Publications journals to adhere to the CREMLS guidelines and checklist to ensure that they have considered and addressed all relevant details for their work before initiating their submission and review process. More complete and high-quality reporting benefits the authors by accelerating the review cycle and reducing the burden on reviewers. Hence, the need exists for reporting guidelines and checklists for papers describing prognostic and diagnostic ML studies. This is expected to assist, for example, in reducing missing documentation on hyperparameters for an ML model and to clarify how data leakage was avoided. We have observed that peer reviewers have, in practice, been asking authors to improve reporting on the same topics covered in the CREMLS checklist. This is not a surprise given that peer reviewers are experts in the field and would note important information that is missing. Nevertheless, we would encourage reviewers to use the checklist regularly to ensure completeness and consistency.

The CREMLS checklist’s scope is limited to ML models using structured data that are trained and evaluated in silico and in shadow mode. This provides a significant opportunity to expand on the CREMLS to different data modalities and additional phases of model deployment. Should such extended reporting guidelines and checklists be developed, they may be considered for recommendation for submissions to JMIR Publications journals, incorporating lessons learned from the initial checklist for studies reporting the use of ML models.

## Conclusion

There is evidence that the completeness of reporting of research studies is beneficial to the authors and the broader scientific community. For prognostic and diagnostic ML studies, many reporting guidelines have been developed, and these have been consolidated into CREMLS, capturing the combined value of the source guidelines and checklists in one place. In this Editorial, we extend journal policy and recommend that authors follow these guidelines when submitting articles to journals in the JMIR Publications portfolio. This will improve the reproducibility of research studies using ML methods, accelerate review cycles, and improve the quality of published papers overall. Given the rapid growth of studies developing, evaluating, and applying ML models, it is important to establish reporting standards early.

## Abbreviations

AI: artificial intelligence
CREMLS: Consolidated Reporting of Machine Learning Studies
EQUATOR: Enhancing the Quality and Transparency of Health Research
ML: machine learning
